# Short‐ and Long‐Term Clinical Outcomes of Preeclampsia in Women and Their Neonates in Pregnancies With and Without HIV Infection

**DOI:** 10.1155/ijhy/9198942

**Published:** 2026-06-21

**Authors:** Charlotte Ramdass, Jagidesa Moodley, Nnabuike Chibuoke Ngene, Nalini Govender

**Affiliations:** ^1^ Department of Basic Medical Sciences, Faculty of Health Sciences, Durban University of Technology, Durban, 4001, South Africa, dut.ac.za; ^2^ Department of Obstetrics and Gynaecology, School of Clinical Medicine, College of Health Sciences, Women’s Health and HIV Research Group, University of KwaZulu-Natal, Durban, South Africa, ukzn.ac.za; ^3^ Department of Obstetrics and Gynaecology, Rahima Moosa Mother and Child Hospital, Johannesburg, Gauteng, South Africa, unicamp.br; ^4^ Department of Obstetrics and Gynaecology, Faculty of Health Sciences, School of Clinical Medicine, University of Witwatersrand, Johannesburg, Gauteng, South Africa, wits.ac.za

**Keywords:** endothelial dysfunction, HIV, hypertensive disorders of pregnancy, long-term adverse effects, preeclampsia

## Abstract

Preeclampsia (PE) is characterized by new‐onset hypertension at or after 20 weeks of pregnancy, associated with either uteroplacental dysfunction and/or maternal organ dysfunction that involves any of the hematological, renal, hepatic, cardiovascular, and central nervous systems. It complicates 2%–8% of pregnancies worldwide and is a significant contributor to maternal and perinatal morbidity and mortality. The multiorgan involvement in PE may extend beyond the immediate puerperium period and significantly impact the health of both affected women and their children in later life. In sub‐Saharan Africa, long‐term data on PE remain limited, irrespective of the substantial HIV burden in the region and the probable interaction between hypertensive disorders of pregnancy, HIV infection, and antiretroviral therapy. Therefore, this narrative review provides an update on the short‐ and long‐term clinical outcomes of PE in the context of HIV infection, for both the mother and affected child. For instance, there is an increased risk of long‐term cardiovascular disease development and renal impairment in the mother. Neonates born from such pregnancies are also at increased risk of low birth weight and the sequelae of prematurity.

## 1. Introduction

In 2020, approximately 287,000 maternal deaths were reported worldwide [1]. The primary causes of these deaths were complications of pregnancy and childbirth, including obstetric hemorrhage, sepsis, hypertensive disorders of pregnancy (HDPs), thromboembolism, and unsafe abortions [1]. HDPs, which affect 5%–10% of pregnancies globally, are the major causes of direct maternal deaths, especially in low‐ and middle‐income countries [2–4]. In South Africa, the latest Saving Mothers report (2023) indicates that obstetric hemorrhages and HDPs are the most common direct causes of maternal deaths, accounting for approximately 16.2% and 16.5% of all maternal deaths, respectively [5]. Typically, HDPs include chronic hypertension, gestational hypertension, preeclampsia (PE), eclampsia, and PE superimposed on chronic hypertension [6]. One of the most frequently reported HDPs is PE, which complicates 2%–8% of pregnancies globally [7]. Annually, approximately 70,000 maternal deaths and 500,000 fetal deaths are attributed to PE [7]. Noteworthy, the incidence of PE is seven times more prevalent in low‐ and middle‐income countries (1.8%–18%) than in high‐income countries (1.3%–6%) [8]. In Africa, the prevalence of PE (including those with and without severe features) has recently been estimated to be between 1.8% and 16.7% [9, 10].

PE is defined as the de novo appearance of hypertension (systolic BP ≥ 140 mmHg and/or diastolic BP ≥ 90 mmHg) at ≥ 20 weeks and 0 days of gestation and with or without any significant proteinuria, maternal organ dysfunction, and/or uteroplacental insufficiency [11]. The new‐onset proteinuria is ≥ 2+ proteinuria on dipstick test, protein: creatinine ratio ≥ 30 mg/mmol, albumin: creatinine ratio ≥ 8 mg/mmol, or ≥ 300 mg protein/24 h [12]. The maternal organ dysfunction includes neurologic complications, pulmonary edema, hematologic complications (platelet count < 150,000/μL, DIC, hemolysis), acute kidney injury (creatinine ≥ 90 μmol/L or ≥ 1 mg/dL), and liver involvement (e.g., elevated aminotransferase levels, such as ALT or AST > 40 U/L) with or without right‐upper‐quadrant or epigastric abdominal pain. The uteroplacental dysfunction includes fetal growth restriction, placental abruption, and angiogenic imbalance [6, 13].

Based on gestational age at diagnosis, PE is clinically recognized as two subtypes: early‐onset PE (EOPE) (< 34 weeks) and late‐onset PE (LOPE) (≥ 34 weeks) [14]. Globally, the rate of EOPE is, however, disproportionately lower than that of LOPE [15]. In a study conducted in the United States of America (Washington) on singleton pregnancies, EOPE accounted for approximately 10% of preeclamptic pregnancies [16]. Of note, EOPE pregnancies are associated with less favorable fetal and maternal outcomes, including intrauterine fetal growth restriction, fetal death due to placental abruption, and iatrogenic prematurity [17]. In contrast, LOPE affects 90% of preeclamptic pregnancies, especially in women with chronic hypertension, thrombophilia, increased BMI, and pre‐existing diabetes mellitus or autoimmune disease [18]. Thus, LOPE is often associated with moderate maternal clinical manifestation and milder adverse perinatal outcomes [17].

There is a paucity of data from low‐ and middle‐income countries in sub‐Saharan Africa regarding the short‐ and long‐term medical impact of PE on women and their affected offspring. Noteworthy, women of reproductive age also account for 52% of all adults living with human immunodeficiency virus (HIV) infections worldwide [19]. Furthermore, pregnant women living with HIV have 2–10 times increased risk of death during pregnancy and the postpartum period compared with uninfected pregnant women [20–22]. Globally, South Africa remains the country with the highest HIV infection rates, with approximately 8 million people living with HIV infection (PLHIV), and almost half of whom are women [19]. Notably, one in three pregnant women receiving antenatal care in South Africa is HIV‐infected [23, 24]. High HIV infectivity in this vulnerable population increases the number of maternal deaths. [25]. In South Africa, for instance, HIV accounts for the highest percentage (17.1%) of all maternal deaths [5].

Of note, PE is associated with deterioration of immune tolerance or immunological incompatibility between the mother and fetus and a consequent hyperinflammatory maternal response, whereas HIV infection correlates with a decline in immune activity [26]. HIV‐associated PE results in neutralization of the immune response. Therefore, dysregulation of the complement system during PE enhances HIV infectivity due to upregulation of immune suppression factors [27]. In a preeclamptic pregnancy, there is a shift from the T helper 2 (Th2) anti‐inflammatory response present in a healthy pregnancy to the T helper 1 (Th1) pro‐inflammatory response [28]. Immune suppression, as observed in untreated HIV infection, may reduce PE development risk in affected women. However, the rollout of antiretroviral therapy (ART) monotherapy and highly active antiretroviral therapy (HAART) reverses the pattern causing a shift from T1 to T2 response [29]. Nucleoside reverse transcriptase inhibitors (NRTIs), non‐nucleoside reverse transcriptase inhibitors (NNRTIs), and protease inhibitors (PIs) decrease endothelial cell proliferation and migration via defective tyrosine kinase receptor and vascular endothelial growth factor‐2 (VEGFR‐2) signaling and enhanced mitochondrial oxidative stress which leads to greater reactive oxygen species generation [30–32]. The use of HAART and/or ART therefore reconstitutes the immune function in affected individuals thereby increasing PE development risk in this vulnerable population [25, 33–35]. In view of the major obstetric clinical issues associated with PE and HIV, it poses a significant public health concern [36]. Due to the high geographic prevalence of both HIV and PE in South Africa, this narrative review describes the subtypes, pathophysiology, clinical manifestations, and risk factors of PE as well as the short‐ and long‐term health outcomes of affected women and their children.

## 2. Risk Factors, Clinical Manifestation, and Management of PE

To date, a plethora of risk factors for PE development have been reported, but the exact cause of the disease has not been identified [37]. Pre‐existing hypertension, chronic kidney disease (CKD), insulin‐dependent diabetes, and a history of EOPE are key risk factors in PE development [38]. However, several inconsistencies, such as a lack of evidence detailing the type, timing, and frequency of assessment modalities, exist in high‐income countries regarding these factors [39]. As a result, the underlying cause of PE remains unclear due to differences among populations and ethno‐geographic groups [40]. This remains a major concern in sub‐Saharan Africa, as the high maternal mortality rates associated with HDPs remain static [1].

In a recent systematic and meta‐analysis review of risk factors for the development of PE and eclampsia conducted in sub‐Saharan Africa (viz., Nigeria, Ethiopia, SA, and Sudan), women in both extremes of reproductive age (≤ 20 and ≥ 35 years) were at significant risk for PE (OR: 7.6; 95% CI: 2.9, 19.9) and eclampsia (OR: 10.2; 95% CI: 3.2, 32.2) development [41]. Moreover, women with a history of PE were approximately 6 times more at greater risk of PE and eclampsia development (OR: 5.6; 95% CI: 1.82, 9.38), compared to those without a history of PE in past pregnancies. Additionally, women with a hereditary predisposition of PE were 1.68 times at greater risk for PE and eclampsia development during pregnancy (OR: 1.68; 95% CI: 1.26, 2.11). Additional findings indicate that women with higher BMI were at 2 times greater risk of PE and eclampsia development compared with women with a healthy BMI (OR: 1.69; 95% CI: 1.17, 2.21). Pregnant women with chronic hypertension were at 2 times higher risk of PE and eclampsia development compared to women without (OR: 2.26; 95% CI: 1.49, 3.03). Women who had anemia during pregnancy were > 3 times more likely to develop PE and eclampsia as compared to those without (OR: 3.22; 95% CI: 2.70, 3.75). However, the association between nutrition‐related factors, antenatal care visits, birth spacing, and the risk of PE development was inconclusive [41]. Furthermore, genetics and epigenetic predisposition have been reported as risk factors with over 15 genes, such as the soluble fms‐like tyrosine kinase‐1 gene (SNP ID: rs12584067) being implicated [42].

The only definite treatment for PE is placental delivery. Again, EOPE is usually severe and associated with shorter gestational periods due to the high possibility of placental abruption, premature delivery, intrauterine fetal growth restriction, and intrauterine fetal death [43, 44]. While LOPE pregnancies extend toward the later weeks of the third trimester and prevent the sequelae of prematurity, it is associated with the maternal acute kidney injury, hepatic failure, hepatic rupture, pulmonary edema, cerebral hemorrhage, disseminated intravascular coagulation, and progression to eclampsia [44]. The management of PE is thus based on the time of diagnosis and the severity of the disease [45]. Data indicate that termination of pregnancy is the best option for both mother and neonate, particularly when the fetus weighs ≥ 1500 g (gestational age ≥ 30 weeks) [46, 47]. However, the timing of delivery of women with PE without severe features varies from 34–37 weeks’ gestation depending on health facility protocols [48, 49]. In some facilities, mild PE diagnosed before 37 weeks’ gestation requires careful clinical monitoring as immediate delivery during this stage escalates neonatal respiratory distress syndrome risk [44]. In PE with severe features, delivery is usually at 34 weeks’ gestation unless the worsening of maternal and/or fetal condition warrants delivery earlier [43]. Of note, PE with severe features is clinically characterized by severe hypertension (≥ 160/110 mmHg), thrombocytopenia (< 100,000 platelets/μL), elevated liver transaminase levels (at least twice the upper limit of normal concentrations), severe persistent right‐upper‐quadrant pain, visual disturbances, persistent severe headaches, and/or acute pulmonary edema [50].

Although PE is distinct from chronic hypertension and gestational hypertension, it can be superimposed on a background of chronic hypertension, as evidenced by uteroplacental dysfunction, new‐onset maternal organ impairment including significant proteinuria [51]. Any other HDPs, such as gestational hypertension, may also progress to PE.

## 3. Pathophysiology of PE

The pathophysiology of PE development is associated with diverse theories [52, 53]. However, abnormal placentation occurs early in pregnancy because of inadequate invasion by fetal cytotrophoblasts to remodel the maternal spiral arteries [54, 55]. This subsequently results in placental ischemic reperfusion which leads to immune dysregulation, increased oxidative stress, and the placental release of inflammatory cytokines and antiangiogenic factors [56–58]. In sub‐Saharan Africa, the high prevalence of HIV infection and ART, which modulate immune and endothelial responses, exacerbates PE pathogenesis [59].

**FIGURE 1:**
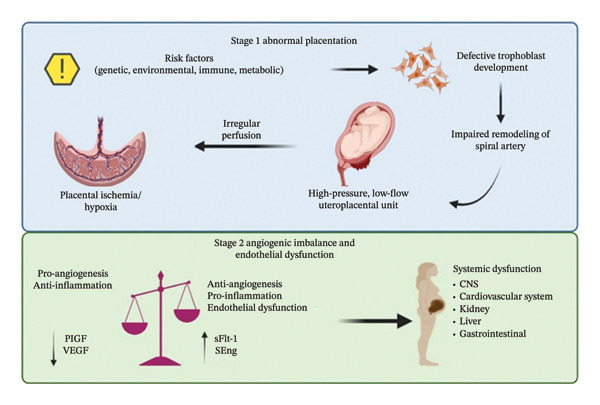
The two‐stage model of preeclampsia development. Stage 1 is the preclinical stage and is characterized by abnormal placentation, which leads to Stage 2, characterized by the angiogenic imbalance and endothelial dysfunction (adapted and modified from [60]). This figure was generated using BioRender (https://biorender.com/).

PE may be characterized by the modified two‐stage model, which involves the abnormal placental development followed by the angiogenic dysregulation (Figure 1).

With regard to the subcategories of PE, EOPE is characterized by abnormal placental development during the first trimester, weeks before the clinical presentation of PE [61, 62]. In contrast, LOPE is characterized by little or no atypical remodeling of the spiral arteries and thus results in minimal intrauterine fetal growth restriction. However, there is endothelial injury resulting in microvascular damage, generalized vasoconstriction, and reduced blood flow to multiple organs. Despite in‐depth research [63–65], the pathophysiology of the LOPE subtype remains poorly understood because most patients present with entities of both EOPE and LOPE [66]. The pathophysiology remains complex with many causes other than vascular remodeling defects, including imbalance between the metabolic needs of the growing fetus close to term and maternal supply [67] and abnormalities of umbilical/uterine artery Doppler velocimetry [67]. Due to the variations in PE subtypes, the mechanisms, such as immune cell activation and consequent immunological memory, could contribute to long‐term maternal consequences [68, 69].

## 4. Short‐Term Clinical Outcomes of PE for Mother and Neonate

**FIGURE 2:**
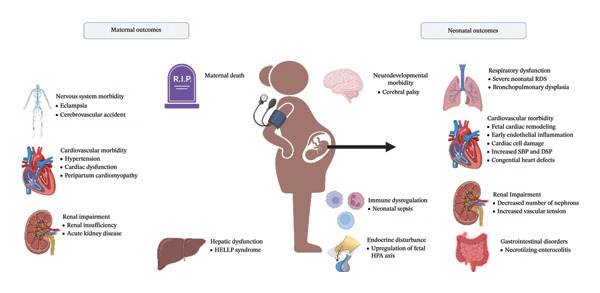
Short‐term clinical consequences of preeclampsia. Short‐term maternal outcomes include maternal death, nervous system morbidity, cardiovascular morbidity, renal impairment, and hepatic dysfunction. Short‐term neonatal outcomes include neurodevelopmental/central nervous system morbidity, cardiovascular morbidity, renal impairment, endocrine disturbance, immune dysregulation, and gastrointestinal disorders. This figure was generated using BioRender (https://biorender.com/).

PE can result in serious maternal health impairments during pregnancy, postpartum, and beyond. The short‐term impact of PE on both the mother and neonate is illustrated in Figure 2. The most severe short‐term maternal clinical outcome of PE includes maternal death [70], severe hypertension, cardiac dysfunction, renal impairment (renal insufficiency, acute kidney injury), hepatic dysfunction (hemolysis, elevated liver enzymes, and low platelet count [71]), and nervous system morbidity, which often progresses to eclampsia and increased risk of a cerebrovascular accident (Figure 2) [60]. The clinical complications may present during the postpartum period or worsen, as this is a high‐risk period due to the transition from continuous to reduced medical monitoring [72]. Patients readmitted postpartum due to PE (without a prior hypertension diagnosis) are thus at high risk of eclampsia, stroke, and death [73].

In South Africa, the short‐term clinical outcomes of PE are compounded by a high prevalence of comorbidities, such as HIV infection and resource‐constrained healthcare settings. Maternal morbidity and mortality rates related to PE remain disproportionately high compared to high‐income countries, partly due to late diagnosis, limited access to specialized care, and challenges in managing complications, such as eclampsia, HELLP syndrome, renal impairment, and cerebrovascular events [55, 74]. According to the 2023 Saving Mothers report, HDPs, including PE, contribute to approximately 16.5% of all maternal deaths in South Africa, with a majority being potentially preventable through timely and adequate clinical intervention [12].

Short‐term effects of PE on the neonate were recently described in a narrative review by Koulouraki et al. [18]. Reported effects include neurodevelopmental and/or central nervous system morbidity, viz., cerebral palsy (CP) [75, 76]; cardiovascular morbidity, such as fetal cardiac remodeling [77], early endothelial inflammation and cardiac cellular injury [78], and elevations in systolic and diastolic blood pressures in neonates [79, 80]; and congenital heart defects [81, 82]. Additional effects reported include renal impairment, viz., decreased number of nephrons, dysregulation of vasoactive compounds, and consequent elevation in renal vascular tension [83, 84]; and an upregulated fetal hypothalamus–pituitary–adrenal axis [85]. Furthermore, PE was associated with respiratory dysfunction and consequent severe neonatal respiratory distress syndrome [73] and bronchopulmonary dysplasia [86–88]. Noteworthy, a lower risk of bronchopulmonary dysplasia was reported in very‐low‐birth‐weight infants exposed to PE [89]. Additional observations include immune dysregulation, such as neonatal [90] and necrotizing enterocolitis, as examples of gastrointestinal disorders [91].

(Figure 2) The underlying mechanism for short‐term neonatal complications emanating from PE remains unclear, but it is postulated that the preeclamptic condition causes epigenetic changes that negatively affect developmental plasticity [18]. Given that congenital heart defects are the most prevalent nonchromosomal congenital anomalies [92], their association with PE is debatable.

## 5. Long‐Term Clinical Outcomes in Women and Their Neonates

PE causes significant long‐term maternal and neonatal complications, which persists beyond pregnancy and the immediate puerperium period [60, 93].

### 5.1. Long‐Term Maternal Outcomes

#### 5.1.1. Cardiovascular Health

Cardiovascular disease (CVD) morbidity and mortality advance throughout a woman’s life [94, 95]. This is evidenced by published data on long‐term CVD development and its associated risk factors post a preeclamptic pregnancy [43, 80, 96, 97], albeit the underlying mechanisms responsible for the association between PE and subsequent CVD development remain debatable. Similar risk factors are noted for PE and obesity, dyslipidemia, insulin resistance, pro‐inflammatory and hypercoagulable state, and endothelial dysfunction [98]. Pregnancy can thus only be a trigger for cardiovascular modifications that manifest in PE development. However, PE and other HDPs may independently contribute to the onset of postpartum CVD by renin–angiotensin–aldosterone system (RAAS) dysregulation and consequent endothelial damage, and/or hyperinflammation [99, 100].

HDPs, such as PE, are the leading risk factors for CVD [101–103]. Long‐term clinical manifestations include chronic hypertension, ischemic heart disease, and stroke [104–106]. Findings from a recent systematic review and meta‐analysis showed that PE is associated with an higher risk of long‐term heart failure (risk ratio [12], 4.19; 95% CI, 2.09–8.38), coronary heart disease (RR, 2.50; 95% CI, 1.43–4.37), CVD death (RR, 2.21; 95% CI, 1.83–2.66), and stroke (RR, 1.81; 95% CI, 1.29–2.55) [107]. Furthermore, long‐term heart failure risk was the highest 1–10 years after the preeclamptic pregnancy (aRR, 8.42; 95% CI, 4.39–16.17) compared with < 1 year (aRR, 4.10; 95% CI, 2.90–5.80) or > 10 years (aRR, 1.60; 95% CI, 0.73–3.50). Moreover, the risk for coronary heart disease (aRR, 3.10; 95% CI, 1.56–6.16) and stroke (aRR, 2.22; 95% CI, 1.73–2.85) was significantly increased within the first 12 months postdelivery compared with other time points. In CVD death, the increase in risk was similar at 1–10 years (aRR, 2.30; 95% CI, 1.65–3.20) and > 10 years (aRR, 2.21; 95% CI, 1.73–2.81) postdelivery [107]. Despite the notable unmeasured confounders observed, these findings highlighted the significance of educating patients about risk and lifestyle modifications to reduce long‐term CVD development and recommended regular monitoring of women with a predisposition for PE.

The risk for future adverse cardiovascular maternal complications after first‐time and recurrent PE and the current use of basic and specialized care post a preeclamptic pregnancy were recently investigated in a large‐scale German cohort study, using claims data from German statutory health insurance [52]. The findings from this report demonstrate that women who experienced PE once were at greater risk for cardiovascular (hazard ratio [HR] [12] = 1.29) or hypertensive (HR = 4.13) events. In addition, women affected by recurrent PE had a higher risk of developing CVD (HR = 1.53) or hypertension (HR = 6.01). Moreover, mothers frequently visited general practitioners but not cardiologists postpartum (0.3% and 2.4%). These findings are indicative that women in this study affected by PE are at increased risk of future chronic hypertension and CVD development than women without, especially those with recurrent PE. This is an important consideration for future medical guidelines as preventive management programs are warranted.

Giorgione et al. report that biochemical and echocardiographic abnormalities do not resolve after a preeclamptic pregnancy and that the majority of preeclamptic women continue to be hypertensive in the immediate postnatal period with some displaying unknown characteristics of cardiac dysfunction [97]. Furthermore, home BP self‐monitoring combined with effective BP control in the immediate postnatal period has long‐term maternal benefits. Therefore, research validating optimal BP measurement methods, frequency, and treatment regimens in the 12 weeks following childbirth is needed. These data are important to improve future cardiovascular health following a preeclamptic birth.

#### 5.1.2. Renal Health

Renal failure, acute kidney injury, and future risk of end‐stage renal disease (ESKD) are often associated with PE [98]. Albeit following delivery and the resolution of the PE symptoms, complete renal recovery does occur in most cases. However, women who experienced previous adverse pregnancy outcomes are at greater risk of developing microalbuminuria, CKD, or ESKD later in life than their healthy counterparts [108, 109]. Findings from a systematic review and meta‐analysis of observational studies involving 273 preeclamptic women and 333 women with uncomplicated, normotensive pregnancies confirm that 31% of preeclamptic women developed microalbuminuria seven years postpartum compared to 7% of normotensive pregnancies [110]. Moreover, the risk of microalbuminuria development was 4 times greater after mild PE and 8 times greater after severe PE.

Other investigators have also reported that adverse pregnancy outcomes, such as PE and premature delivery, may cause premature vascular aging and permanent damage and a greater risk of kidney disease and CVD [111]. A large retrospective observational database study using claims data from the AOK Baden‐Wuerttemberg health insurance registry in Germany was recently conducted to assess the risk of CKD development in women who had prior preterm delivery in the synergy of PE [112]. After adjusting for maternal age, diabetes, obesity, dyslipidemia, and different CKD stages in a cohort of 193,152 women, 6.58% preterm deliveries (16,948) and 6.61% births with ≥ 1 previous diagnosis of PE (14,448) were reported. Moreover, 1821 women in the cohort developed CKD; women with ≥ 1 previous preterm delivery (HR = 1.789) and women with PE (HR = 1.784) had greater risk for CKD development, compared to those without risk exposure. Furthermore, the highest risk for CKD development was in women who had previous preterm birth and PE (HR = 5.227). These effects were similar only for mild to moderate CKD, but were strongly increased for severe CKD (HR = 11.90). Findings from this study suggest that preterm birth and PE are independent risk factors for all CKD stages, and their dual exposure increased the maternal risk of CKD development in the first decade after pregnancy.

ESKD requires long‐term dialysis or renal transplantation [98] for a better prognosis. Data from a recent systematic review and meta‐analysis study involving 23 studies and 5,769,891 participants demonstrated a higher risk of ESKD among women with PE [109]. Moreover, PE was significantly associated with a greater risk of CKD (pooled adjusted risk ratio [aRR]: 2.11; 95% CI, 1.72–2.59), ESKD (aRR: 4.90; 95% CI, 3.56–6.74), and kidney‐related hospitalization (aRR: 2.65; 95% CI, 1.03–6.77). Additionally, EOPE was linked to enhanced risk of ESKD (aRR, 5.66; 95% CI, 3.06–10.48) [109], although it remains unclear whether adverse pregnancy outcomes emanated due to an existing predisposition toward kidney disease or as a result of induced endothelial and/or organ injury. Thus, ensuring an optimized long‐term follow‐up and implementing preventive interventions to reduce the risk of kidney disease development are essential for this population.

#### 5.1.3. Metabolic Health

A woman’s pre‐pregnancy metabolic and endothelial health influences her vulnerability to PE development and future risk of metabolic and CVD [113]. Preeclamptic women are more likely to be overweight [114], have higher lipid levels [115] and BP [114], as well as are more likely to experience insulin resistance [116] and thrombophilia compared with women with a normotensive pregnancy [117]. In a longitudinal cohort study, 107 previously diagnosed preeclamptic women were assessed for traditional risk factors of metabolic syndrome, viz., insulin resistance, obesity, dyslipidemia, hypertension, and microalbuminuria, at 3–30 months postparturition (visit 1) and 24–65 months later (visit 2) [118]. The data highlight that at visit 1, 9% (*n* = 10) previously diagnosed preeclamptic women had metabolic syndrome, whereas at visit 2, 13% (*n* = 14) were diagnosed. Among those previously diagnosed with PE, metabolic syndrome resolved in 3% (*n* = 3) over time, while 7% (*n* = 7) developed persistent metabolic syndrome.

Findings from a systematic review and meta‐analysis investigating the association between PE and eclampsia on subsequent metabolic and biochemical outcomes revealed significant elevations in systolic and diastolic BP, C‐reactive protein, glucose, insulin, HOMA‐IR index, total cholesterol, LDL, triglycerides, BMI, waist, waist‐to‐hip ratio, weight, and the risks of hypertension and MetS among women with PE/eclampsia (*n* = 3300) compared to healthy individuals (*n* = 13, 967) who were tracked since 3 months postdelivery up to 32 years postpartum [71]. The data demonstrate compelling evidence that correlates PE and eclampsia with an increased risk of future metabolic complications and consequent cardiovascular and endocrine disease among affected women. The association between PE and maternal BMI, and other indicators of metabolic syndrome, including high BP (> 130/80 mmHg), impaired fasting glucose (≥ 100 mg/dL), high triglyceride levels (> 150 mg/dL), and low HDL levels (< 50 mg/dL), places preeclamptic women at risk of future type 2 diabetes mellitus [113]. PE is reported to be independently associated with enhanced maternal future risk of diabetes mellitus development, even after adjusting for BMI or the presence of gestational diabetes [119]. However, after the exclusion of women with gestational diabetes, the risk of diabetes development was moderately increased in preeclamptic women [120].

Findings from a systematic review and meta‐analysis study evaluate the association of previous HDPs (PE or gestational hypertension) with incident diabetes; 16 cohort studies revealed a significantly higher risk of subsequent diabetes in women with any previous HDP (HDP: adjusted hazard ratio [aHR] [106] 2.24, 95% CI 1.95, 2.58) from a total of 3,095,457 participants [121]. Moreover, in this cohort (i.e., unspecified HDPs, *n* = 5; PE, *n* = 4; gestational hypertension and PE, *n* = 7), the risks of subsequent diabetes were significantly higher in women with a history of gestational hypertension (aHR 2.19 [95% CI 1.69, 2.84]), PE (aHR 2.56 [95% CI 2.02, 3.24]), and EOPE (aHR 3.05 [95% CI 2.05, 4.56]). Of note, in studies that excluded women with gestational diabetes mellitus, there was a continuity in the association between HDPs and diabetes (aHR 2.01 [95% CI 1.77, 2.28]). This is indicative that HDPs are independently associated with an increased risk of diabetes and thus require further studies to establish how HDP contributes to diabetes risk prediction, for the development of evidence‐based screening and prevention strategies.

### 5.2. Long‐Term Outcomes in Children

#### 5.2.1. Cardiometabolic Health

Experimental and epidemiological evidence based on the Barker theory suggests that maternal hypertension or placental ischemia increases the risk of hypertension, CVD, and stroke in affected neonates [17, 111, 122, 123]. In a recent review of observational and interventional studies, PE influenced the neonatal cardiovascular system independent of preterm birth and is related to endothelial dysfunction, increased carotid intima media thickness, and reductions in cardiac function that cannot be attributed to prematurity alone [124]. This was corroborated by Koulouraki et al. who confirmed that adverse changes in neonatal left and right ventricular structure and function are independent of preterm birth [18]. Furthermore, the lasting effects of PE on the neonatal cardiovascular system were correlated to endothelial injury, angiogenic imbalance, smooth muscle abnormalities, and undetectable metabolic disorders in the mother, as likely underlying mechanisms were involved.

Neonates born to preeclamptic pregnancies are reported to have a higher risk of developing high BP and 2 times the risk of future stroke [125, 126]. Moreover, in utero exposure to PE was associated with a 2.39 mmHg higher systolic (95% CI: 1.74–3.05; *p* < 0.0001) and a 1.35 mmHg higher diastolic BP (95% CI: 0.90–1.80; *p* < 0.00001) during childhood (4–10 years of age), while BMI was increased by 0.62 kg/m^2^ (*p* < 0.00001) [127, 128]. In a prospective U.S. high‐risk cohort study conducted in 754 mother–child pairs, PE was associated with 5.34% higher offspring systolic BP (95% CI, 1.37–9.30), from early childhood to adolescence, as evidenced by cord blood vitamin D deficiency [129]. More recently, findings from a systematic review and meta‐analysis involving 23 studies in Asia, Europe, Oceania, and the Americas established a greater risk of high BP among preeclamptic offspring during childhood and early adulthood [130]. Moreover, systolic BP was 2.0 mmHg higher (95% CI: 1.2, 2.8), and diastolic BP was 1.4 mmHg higher (95% CI: 0.9, 1.9) in offspring exposed to PE in utero, compared to those born to healthy mothers. It is possible that environmental and genetic factors, such as maternal or neonate BMI, life environment, ethnicity, and dietary and nutritional considerations, may be involved.

In Korea, Yang and coworkers compared the frequency of early childhood obesity (20–30 months) between PE and non‐PE‐affected pregnancies [131]. The incidence of low‐birth‐weight (LBW) neonates was higher in the PE group (*n* = 29,710) compared to the non‐PE group (*n* = 1,533,916) (24.70% vs. 3.33%, *p* < 0.01). Furthermore, BMI was significantly greater in PE‐affected offspring than in non‐PE‐affected offspring. After adjusting for maternal age, maternal pre‐pregnancy diabetes mellitus or hypertension, primiparity, cesarean section, preterm birth, neonatal sex, and birth weight, the obesity risk was higher in PE‐affected offspring (odds ratio [OR] = 1.34, 95% confidence interval = 1.30–1.38), while BMI and obesity frequency were higher during early childhood in the PE‐affected offspring, regardless of a higher proportion of LBW. It is plausible that the multifarious interplay of various mechanisms, such as genetic background and environmental conditions, may be involved [132].

#### 5.2.2. Neurodevelopmental Health

PE is linked to increased risk of future neurodevelopmental disorders, such as autism spectrum disorder (ASD), attention‐deficit/hyperactivity disorder (ADHD), and CP [18]. Although PE is one of the leading causes of adverse neurodevelopmental obstetric outcomes [18, 133], the association was only recently reported as being independent of its effects on gestational age and birth weight [134]. The causal mechanisms through which PE exerts these effects remain unclear; however, inflammation and oxidative stress, both of which affect maternal, placental, and fetal circulation, are implicated [18]. It is possible that these mechanisms expose the fetus to maternal immune activation and elevated inflammatory cytokine concentrations.

ASD is defined by repetitive behaviors and communication, social behavior, and sensory processing challenges, and is usually diagnosed during early childhood [135]. The etiology is multifactorial and is inclusive of both environmental and genetic factors [136]. PE is reported as an independent risk factor for ASD in several cohort studies [137–139]. Findings from a U.S. retrospective population‐based clinical cohort study investigating 364,588 mother–child pairs (PE‐exposed group [*n* = 16,205]; PE‐unexposed group [*n* = 348,383]) of singleton births between 2001 and 2014 showed a significant link between PE and ASD risk in children, with enhanced risk with EOPE [140]. Noteworthy, 2.9% (465/16,205) of children diagnosed with ASD by the age of 5 years were born from preeclamptic pregnancies, while 1.9% (*n* = 6729/348,383) were born from non‐preeclamptic pregnancies, with an HR of 1.36 (95% CI 1.23–1.49) of ASD risk for preeclamptic‐exposed versus non‐preeclamptic groups. Moreover, EOPE was associated with higher ASD risk (*p* = 0.003), with HRs of 1.62 (95% CI 1.33–1.98), 1.43 (95% CI 1.20–1.69), and 1.23 (95% CI 1.08–1.41), for onset at < 34, 34‐37, and ≥ 37 weeks, respectively, relative to the unexposed group.

Approximately 5%–8% of children globally are diagnosed with ADHD probably because of maternal PE [141]. A systematic review and meta‐analysis of nine studies demonstrated an OR of 1.31 for ADHD in children exposed in utero to PE [134]. In another systematic review and meta‐analysis, intrauterine exposure to PE increased the risk of ADHD by 29% (pooled OR of 1.29 [95% CI: 1.20, 1.38]), ASD by 27% (pooled OR of 1.27 [95% CI:1.22, 1.32]), and epilepsy by 35% (pooled OR of 1.35 [95% CI: 1.12, 1.63]) compared to children born to normotensive women [142]. CP results from abnormal development of the fetal or infantile brain and affects approximately 1.5%–3% of children globally [143]. PE and in particular EOPE are associated with preterm birth and fetal growth restriction, and these adverse outcomes increase the risk of CP [75]. The association between PE and the risk of CP in children was investigated using a systematic review and meta‐analysis of 10 studies [76]. However, the findings highlight a lack of statistical significance regarding the relationship between PE and CP pooled (OR = 1.16, 95% CI: 0.77–1.74), and neither was PE associated with CP independently of gestational age. In a prospective, population‐based Norwegian cohort study of 980,560 children born at term (mean [SD] gestational age, 39.8 [1.4] weeks) with a mean (SD) follow‐up of 14.0 (5.6), 2.9% (28,068) were exposed to PE [144]. Furthermore, children born from preeclamptic mothers had an increased risk of ADHD (adjusted OR, 1.18; 95% CI, 1.05–1.33), ASD (adjusted OR, 1.29; 95% CI, 1.08–1.54), epilepsy (adjusted OR, 1.50; 95% CI, 1.16–1.93), intellectual disability (adjusted OR, 1.50; 95% CI, 1.13–1.97), and CP (adjusted OR, 1.30; 95% CI, 0.94–1.80), respectively.

## 6. Long‐Term Maternal and Neonatal Clinical Outcomes of HIV‐Associated PE

### 6.1. Maternal Consequences

**FIGURE 3:**
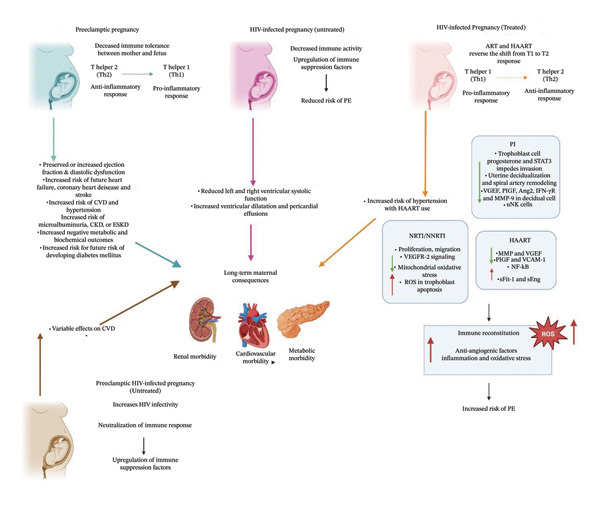
Long‐term clinical outcomes of preeclampsia in HIV‐infected and HIV‐uninfected pregnancies. This figure was generated using BioRender (https://biorender.com/).

The quality of life and life expectancy for PLHIV have markedly improved due to HAART use [145]; however, the risk of acquiring other comorbidities or dying from noncommunicable diseases is increased [146]. The long‐term clinical consequences of PE in HIV‐infected and HIV‐uninfected pregnancies are summarized in Table 1 and Figure 3.

**TABLE 1:** Summary of long‐term clinical outcomes in HIV‐infected and HIV‐uninfected preeclamptic women.

Clinical outcome	HIV‐infected preeclampsia	HIV‐uninfected preeclampsia
Cardiovascular health	Endothelial injury from HAART [147–149]. Increased risk of systolic dysfunction and dilated cardiomyopathy [150].	Twofold to fourfold increased risk of future hypertension, ischemic heart disease, heart failure, and stroke; the risk is comparable to that of traditional risk factors, such as smoking [151].
Renal health	Increased risk of renal impairment, compounded by HIV nephropathy and antiretroviral treatment toxicity [152].	Increased risk for later‐life albuminuria, reduced eGFR, and CKD; the risk is dose‐dependent (higher risk with severe and/or recurrent PE) [152].
Metabolic health	ART may worsen dyslipidemia and insulin resistance [98]. Some ART regimens, viz., older protease inhibitors, increase risk of lipodystrophy and metabolic complications [153]	Twofold increased risk of developing type 2 diabetes and metabolic syndrome [118, 154].

#### 6.1.1. CVD

Pregnancies complicated by both PE and HIV are vulnerable to CVD [155]. PIs and HAART exert an overall negative effect on the CVS and correlate with an increase in cardiac apoptosis, total cholesterol, low‐density lipoproteins, triglycerides, and endothelial dysfunction, a reduction in repair mechanisms and cardiac ATP production, as well as block hyperplasia/hypertrophy [156]. In contrast, integrase strand transfer inhibitors (INSTI), NRTIs, and NNRTIs exert both positive and negative effects on cardiovascular health [156]. Continued use of HAART may lead to endothelial injury due to reduced vasodilation, vasoconstriction, and prothrombosis [147–149]. Approximately 60% of studies from a recent systematic review revealed an association between persistently elevated biomarkers of endothelial dysfunction and a pro‐inflammatory state in PLHIV on HAART [157]. Notably, an improvement was observed in endothelial biomarkers in PLHIV on HAART, which may be attributed to the therapeutic effects of HAART [157].

PE is associated with preserved or increased ejection fraction and diastolic dysfunction and greater risk of hypertension, ischemic heart disease, and stroke postpregnancy [151], while HIV is associated with systolic dysfunction and reduced ejection fraction heart failure with a dilated cardiomyopathy [150]. Transthoracic echocardiography was used to assess and monitor hemodynamics and cardiac structural changes in women with PE (*n* = 15) and women without PE (*n* = 20), as well as in HIV‐positive (*n* = 30) and HIV‐negative (*n* = 40) term pregnancies in sub‐Saharan Africa [159]. These investigators demonstrated minimal changes in cardiac structure and preserved systolic function with diastolic dysfunction in women with PE compared to women without PE. In HIV‐positive pregnant women, a reduction was noted in left and right ventricular systolic function; ventricular dilatation and pericardial effusions were increased compared to HIV‐negative pregnancies. Although this study established echocardiographic reference ranges for specific populations, viz., healthy vs preeclamptic women; and HIV‐positive vs HIV‐negative term pregnancies, it was unable to provide insights on cardiac function in preeclamptic women coinfected with HIV‐1. Thus, it remains unclear whether these findings may be related to the disease itself, disease treatment, or the impact of pregnancy on the cardiovascular system.

Limited data are available on the cardiac function of preeclamptic women living with HIV, and whether cardiac dysfunction is intensified by dual pathology. Additionally, it is unclear whether the severity of PE or CD4 count plays a role in the intensity of cardiac manifestations. There is also no clear consensus on whether HIV treatment ameliorates or exacerbates CVD manifestations [158]. The pathogenesis of HIV infection, PE, and CVD is interconnected and thus augments and perpetuates the disease [158]. Both HIV infection and PE affect the cardiovascular system and present as cardiac dysfunction. Further elucidation of the underlying pathophysiological mechanisms associated with these disease entities is required to provide a framework for the development of novel therapeutic interventions and the development of predictive models to measure development and impact to improve quality of life and decrease disease burden.

#### 6.1.2. Renal Impairment

Acute kidney injury persists during and after the postpartum period in women with PE, HELLP syndrome, and HIV infection [159]. The RAAS and the vasoconstrictor angiotensin II receptors are responsible for increased BP in PE as well as insulin resistance in HIV infection [84]. The RAAS amplifies insulin resistance through various mechanisms, for example, via the effects of angiotensin II [160], aldosterone [161], and damaged pancreatic beta cells [162]. In the case of HIV infection, the elevation in renin production from immune cells activates the RAAS [163], as well as HIV replicon via the renin signaling cascade and cleavage of the HIV Gag polyproteins [164].

The prevalence of acute renal injury, chronic renal diseases, and renal treatment–related toxicity is high in both preeclamptic [165] and HIV‐infected patients [159]. However, the relationship between HIV treatment (HAART, PIs, and/or NRTIs) and RAAS remains unclear [59]. Findings from a hospital‐based cross‐sectional analytical study evaluating the relationship between HAART and hypertension in 315 PLHIV in Cameroon (*n* = 228 were HAART and *n* = 87 were HAART‐naïve) showed that the incidence of hypertension in the HAART group was 36.44% (*n* = 82, CI: 30.15%–43.10%) compared to 13.33% in the HAART‐naïve group (*n* = 12, CI: 7.08%–22.13%, *p* = 0.01), with an OR of 3.86 (95% CI: 1.98–7.50) in the HAART‐treated versus the HAART‐naïve group [166]. Specific regimens showed positive associations with hypertension for TDF/3TC/EFV (OR = 2.83), AZT/3TC/NVP (OR = 2.82), AZT/3TC + EFV (OR = 3.48), and TDF/3TC + NVP (OR = 2.36), whereas AZT/3TC + ATV/r (OR = 0.84) and TDF/3TC + ATV/r (OR = 0.45) were unrelated [167]. The data emphasize a significant association between HAART and hypertension, suggesting that PLHIV on HAART have greater risk of hypertension compared to those untreated. Noteworthy, pregnant women living with HIV may be protected against PE development but may develop the disease once ART is initiated. The underlying mechanism responsible for the effect of HAART on RAAS gene expression and RAAS genetic variants is not fully understood and requires further elucidation [59]. In a study investigating the impact of PE and HIV infection on renal disease, HIV‐1 infection and PE result in endothelial dysfunction in the presence of both diseases [159]. Additionally, HIV‐1 accessory and matrix proteins contribute to increased oxidative stress, apoptosis, and the secretion of pro‐inflammatory cytokines, cell adhesion molecules, and angiogenic molecules during PE, thereby affecting downstream signaling pathways.

#### 6.1.3. Neonatal Consequences

LBW is defined as birth weight less than 2500 g, usually a consequence of either preterm birth or intrauterine fetal growth restriction [168], frequently associated with PE pregnancies [69]. There is a high incidence of LBW in low‐income countries (14% in sub‐Saharan Africa and 25% in South Asia) versus high‐income countries (< 8% in Europe and North America) [169, 170]. LBW is an indication of poor maternal–fetal nutrition and results in various short‐ and long‐term consequences for the neonate including death during infancy, weaker immunity and future growth, and cognitive and neurologic deficits [171, 172]. Earlier studies have linked LBW with increased risk of developing coronary heart disease and diabetes mellitus later in adulthood [173, 174]. A significant association was also reported between maternal HIV infection and LBW in North America [175] and Africa [176, 177]. However, a lack of statistical significance was noted in birth weight between neonates born to HIV‐positive and HIV‐negative women in India [178], Italy [179], and Africa [180, 181]. These findings are indicative that the association between LBW and maternal HIV infection, especially if complicated by PE, is unclear.

Xiao et al. conducted a systematic review and meta‐analysis on 52 cohort studies of women living with HIV and healthy women to assess the association between maternal HIV infection and LBW [181]. Of the studies evaluated, 15,538 and 200,896 women living with HIV were assessed for LBW and prematurity, respectively. The maternal HIV infection was significantly associated with both LBW (pooled OR: 1.73, 95% CI: 1.64, 1.82, *p* <  0.001) and prematurity (pooled OR: 1.56, 95% CI: 1.49, 1.63, *p*  <  0.001). Furthermore, HIV‐infected women were at slightly greater risk of LBW in developing countries compared with women in developed countries (OR: 2.12 [95% CI: 1.81, 2.48] vs. 1.75 [95% CI: 1.44, 2.12]). Additionally, antiretroviral drug usage did not significantly affect the impact of maternal HIV exposure on LBW and prematurity. No significant difference was observed for the association between maternal HIV infection and adverse pregnancy effects, and ARVs reduce the risks of LBW or preterm delivery linked to maternal HIV exposure. We note that considerable heterogeneity exists among these original studies, with the associations across different settings being variable.

## 7. Conclusion

The data from this review suggest that PE predisposes affected women to increased risk of future metabolic and CVD, renal dysfunction, and ESKD. Neonates born to preeclamptic pregnancies are at increased risk of long‐term cardiometabolic and neurodevelopmental outcomes. Given the multifactorial pathophysiology of PE and etiological variations across ethno‐geographic populations, understanding the underlying mechanisms is essential. Such awareness is important for developing long‐term prevention strategies and evidence‐based management guidelines. The clinical consequences and underlying mechanisms of PE in the context of HIV infection are complex and uncertain. In conclusion, the high burden of PE and HIV infection in sub‐Saharan Africa highlights the need for greater clarity of the major pathogenic pathways, such as angiogenic imbalance and endothelial dysfunction, which are regulated in this situation. Understanding how ART affects immune reconstitution in preeclamptic women is necessary for improving maternal, fetal, and child health outcomes.

## Funding

This research was funded by the National Research Foundation (grant number SRUG2205056757).

## Conflicts of Interest

The authors declare no conflicts of interest.

## Data Availability

The data used to support the findings of this study are available from the corresponding authors upon reasonable request.
